# Refractory postoperative eczematous dermatitis after total auricular reconstruction, temporally associated with povidone–iodine: a case report

**DOI:** 10.3389/fped.2026.1787946

**Published:** 2026-03-24

**Authors:** Shigui Lu, Xingyu Xia, Chuanzhi Wang, Jinlong Zou, Weimin Shen

**Affiliations:** Department of Burn and Plastic Surgery, Children’s Hospital of Nanjing Medical University, Nanjing, China

**Keywords:** Nagata technique, pediatric, postoperative eczematous dermatitis, tacrolimus, total ear reconstruction

## Abstract

A 9 year and 4 month old female patient underwent stage I and stage II total auricular reconstruction using the Nagata technique. Following each procedure, she developed localized postoperative eczematous dermatitis involving the reconstructed auricle (and, after stage II, also the abdominal donor site), most consistent with contact dermatitis (allergic vs. irritant) on compromised postoperative skin and temporally associated with local povidone–iodine exposure. Initial management with saline cleansing/irrigation, antihistamines, topical therapy, and systemic antibiotics/corticosteroids produced minimal improvement. Two short, tapering courses of oral tacrolimus were therefore administered (1 mg twice daily initially; total course 17 days for each episode), with clinical improvement noted by day 5 and complete clearance by day 13 during the first course, and improvement by day 3 with complete clearance by day 10 during the second course. Blood pressure and renal/hepatic function were monitored on tacrolimus day 5 and day 10 during both courses and remained within normal limits; tacrolimus trough levels were not measured. No adverse events or infections occurred, and no recurrence was observed at 3-month follow-up.

## Introduction

Autologous rib cartilage–based total auricular reconstruction remains the most widely accepted surgical approach for the treatment of microtia (1). In addition to surgical technique, postoperative complications play a critical role in determining the final aesthetic outcome (2). Among these complications, eczematous dermatitis of the reconstructed auricle is clinically significant yet relatively underreported. Because the skin envelope lies directly over the cartilage framework with minimal intervening soft tissue, uncontrolled eczematous dermatitis may rapidly progress to secondary infection, skin necrosis, framework exposure, and unfavorable scarring, ultimately compromising reconstructive results.

Conventional management of postoperative eczematous dermatitis typically includes systemic antihistamines, antiallergic medications, topical or systemic corticosteroids, and saline irrigation (3). However, reports addressing refractory cases unresponsive to these standard therapies are scarce. Here, we describe a pediatric patient with severe, recurrent postoperative eczematous dermatitis following staged total auricular reconstruction that was successfully treated with oral tacrolimus.

## Clinical report

A 9 year and 4 month old female patient (weight 54 kg; height 152 cm) presented with a 1-day history of right periauricular eczematous dermatitis. She had undergone stage I total auricular reconstruction using the Nagata technique with autologous costal cartilage on July 4, 2023. The immediate postoperative course was uneventful, and the patient was discharged in stable condition.

**Figure 1:**
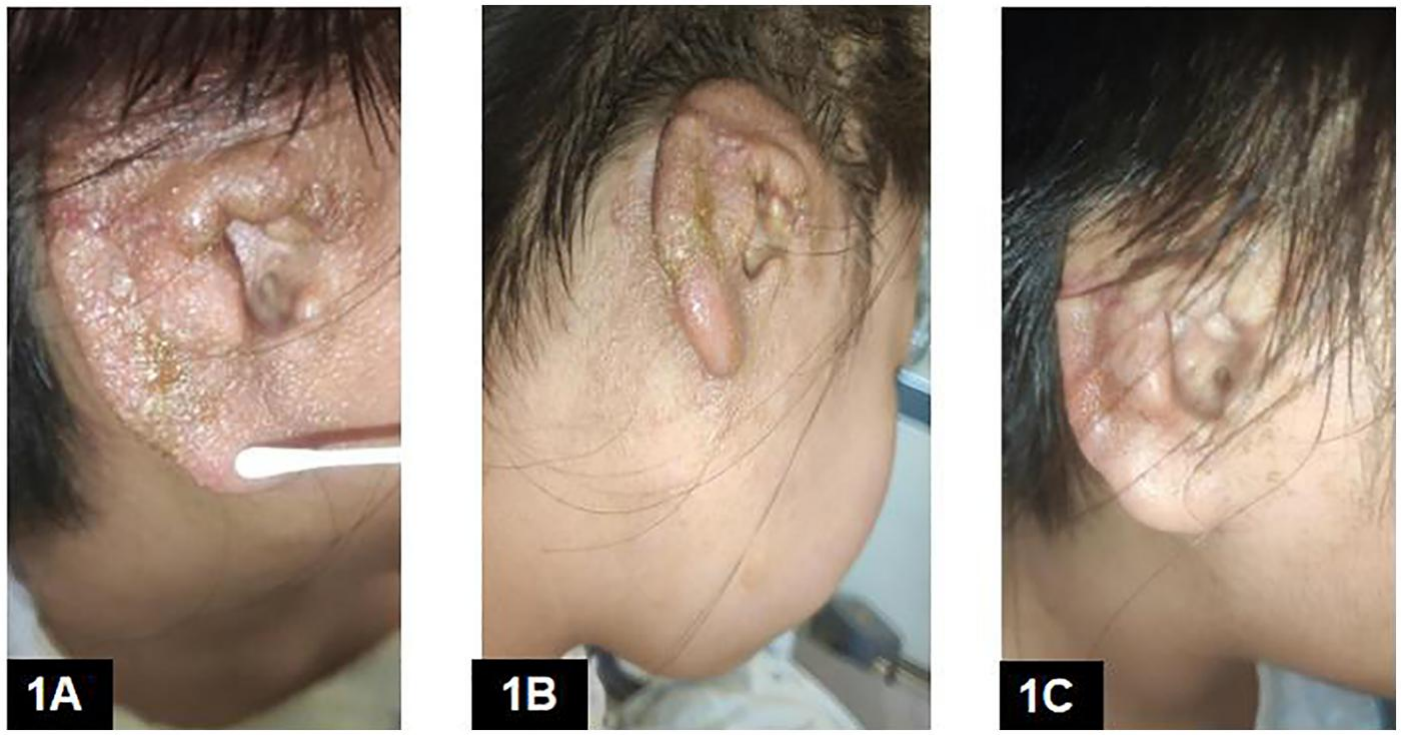
(Stage I postoperative eczematous dermatitis course): **(A)** ∼postoperative week 4, before tacrolimus: periauricular eczematous dermatitis developing after povidone-iodine disinfection (suspected trigger based on temporal association). **(B)** After 7 days of conventional intravenous methylprednisolone, amoxicillin–clavulanate, and saline irrigation, before tacrolimus. **(C)** ∼10 days after initiation of oral tacrolimus therapy.

One month after discharge, erythema and vesicles developed around the reconstructed auricle, accompanied by mild pruritus. The patient had no history of atopic dermatitis or other allergic diseases and no known drug or contact allergies; there was no family history of hereditary skin disorders among first-degree relatives. Physical examination showed no involvement of other body sites, and the eruptions at the reconstructed ear and abdominal donor site were morphologically similar. She had no fever, malaise, or other systemic symptoms. The condition was initially misdiagnosed as infection at a local facility and treated with topical povidone–iodine, after which the lesions rapidly worsened, progressing to diffuse eczematous dermatitis with exudation and crusting involving the entire reconstructed auricle (Figure 1A).

The patient was referred to our department and diagnosed with postoperative eczematous dermatitis most consistent with contact dermatitis (allergic vs. irritant) on compromised postoperative skin. She was treated with intravenous amoxicillin–clavulanate and intravenous methylprednisolone, along with saline irrigation, for 7 days. Despite this regimen, the eczematous lesions persisted (Figure 1B). Given the risk of progression to skin necrosis and cartilage framework exposure, we considered escalation options. Topical tacrolimus was considered; however, the lesions were in an actively oozing/erosive phase, and we were concerned that repeated topical applications could prolong maceration and maintain a moist wound environment, potentially increasing the risk of wound breakdown and framework exposure. Caregivers also reported heightened anxiety because topical preparations made it difficult to visually assess day-to-day changes in the eruption. After shared decision-making, oral tacrolimus was initiated at 1 mg twice daily (total daily dose 2 mg; approximately 0.037 mg/kg/day) for 5 days, followed by 1 mg once daily for 5 days, 0.5 mg once daily for 3 days, and 0.5 mg every other day for 4 days, then discontinued (total course 17 days). Blood pressure and renal/hepatic function were monitored on tacrolimus day 5 and day 10 and remained within normal limits. Tacrolimus trough levels were not monitored. No adverse or unexpected events occurred during treatment. Clinical improvement was first noted after 5 days of oral tacrolimus, and complete clearance was achieved by day 13. No residual dyspigmentation, scaling, or scarring was observed (Figure 1C).

**Figure 2:**
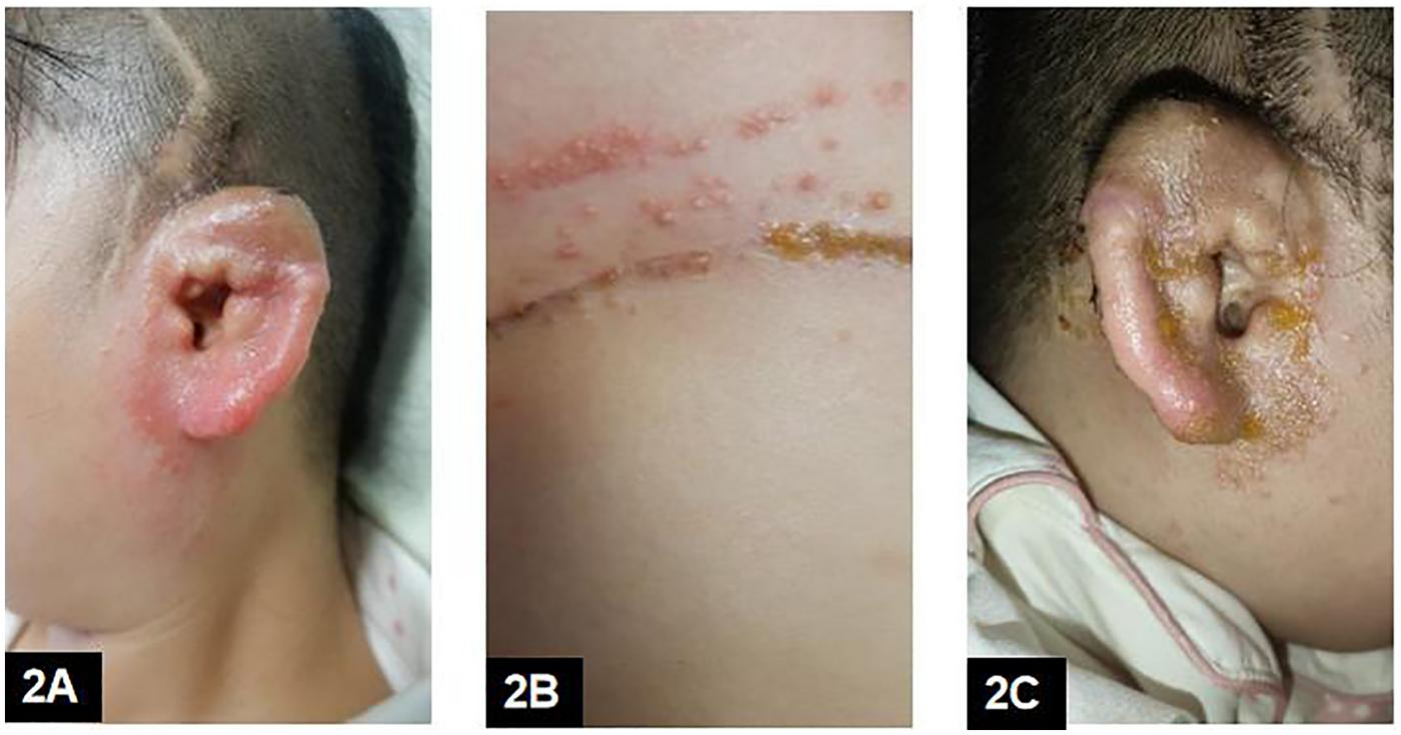
(Stage II early postoperative period): **(A,B)** postoperative day 10, before tacrolimus: recurrent eczematous dermatitis involving the reconstructed auricle **(A)** and abdominal donor site **(B)** shortly after povidone-iodine disinfection during suture removal (suspected trigger based on temporal association). **(C)** Minimal improvement of periauricular eczematous dermatitis after 3 days of conventional treatment (normal saline cleansing, oral loratadine, and combined mometasone furoate/fusidic acid cream), before tacrolimus.

On July 5, 2024, the patient was readmitted for stage II auricular reconstruction. Ten days postoperatively, povidone–iodine was again used for disinfection during suture removal. Shortly thereafter, eczematous dermatitis recurred around the reconstructed auricle and at the abdominal donor site, presenting as erythema, vesicles, and pruritus (Figures 2A,B). Conventional therapy consisting of saline cleansing, oral loratadine, and topical mometasone furoate combined with fusidic acid produced minimal improvement (Figure 2C).

**Figure 3:**
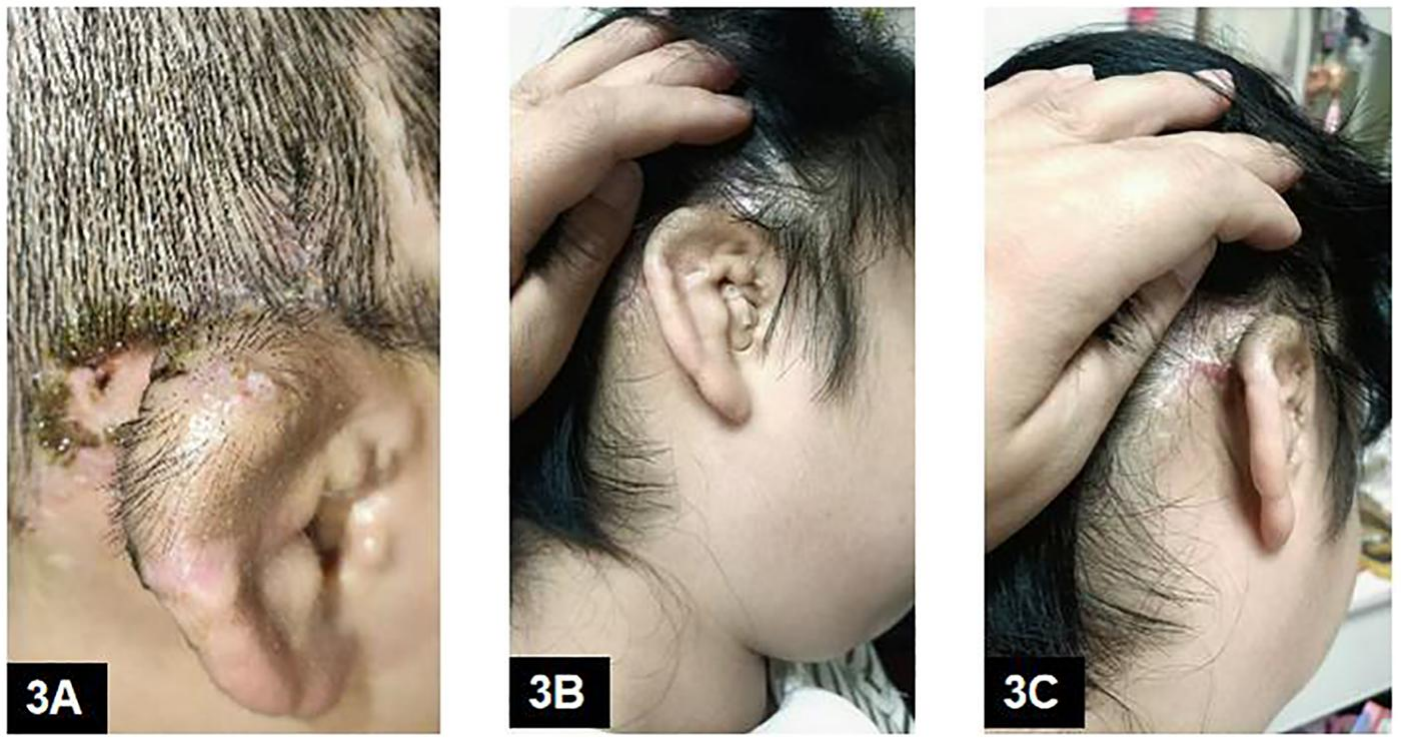
(Response to oral tacrolimus and long-term follow-up after stage II): **(A)** Marked regression of periauricular eczematous dermatitis 3 days after re-initiation of oral tacrolimus 1 mg twice daily. **(B,C)** Three-month follow-up after stage II surgery showing complete remission and excellent aesthetic morphology of the reconstructed auricle.

Based on the previous refractory response, oral tacrolimus was restarted at 1 mg twice daily, using the same total duration and tapering schedule as the first course (total course, 17 days). During the second course, noticeable improvement was observed by day 3 of oral tacrolimus (Figure 3A), with complete clearance by day 10. Blood pressure and renal/hepatic function were again monitored on tacrolimus day 5 and day 10 and remained within normal limits; tacrolimus trough levels were not measured. At 3-month follow-up, no pigmentary or textural sequelae were noted, and the reconstructed auricle maintained an excellent aesthetic appearance (Figures 3B,C).

### Diagnostic assessment

The working diagnosis was postoperative eczematous dermatitis most consistent with contact dermatitis (allergic vs. irritant) on compromised postoperative skin, temporally associated with local antiseptic exposure (including povidone–iodine). Differential diagnoses included postoperative bacterial infection/cellulitis, impetiginized eczematous dermatitis, viral vesicular eruption (HSV/VZV), drug-related exanthem, atopic dermatitis flare, and reactions to dressings or other topical agents. Primary infection was considered less likely given the absence of systemic symptoms and persistence despite systemic antibiotics, although secondary impetiginization could not be excluded. HSV/VZV was considered less likely due to the absence of significant pain, grouped vesicles, or a dermatomal distribution. A generalized drug eruption was considered less likely because both episodes were localized to postoperative fields and recurred after local antiseptic exposure. Patch testing, skin biopsy, and microbiological cultures were not performed; therefore, the etiologic role of povidone–iodine cannot be confirmed.

A CARE-style timeline summarizing the clinical course, co-interventions, and outcomes is provided in Table 1.

**Table 1:** Timeline of the patient's clinical course and treatment.

Date/Postop time	Findings	Suspected trigger/exposure	Treatments (dose/route/duration)	Response/outcome	Figure
July 4, 2023	Stage I total auricular reconstruction (Nagata technique)	Intraoperative antisepsis (povidone-iodine)	Standard postoperative care	Uneventful immediate recovery; discharged stable	—
∼Postop week 4 (1 month after discharge)	Periauricular erythema + vesicles around reconstructed auricle; mild pruritus; no fever/malaise	Topical povidone-iodine applied locally (temporal association)	Topical povidone-iodine (at local facility)	Rapid worsening → diffuse eczematous dermatitis with exudation/crusting involving entire reconstructed auricle	Figure 1A
Next 7 days (after referral to our department)	Persistent exudative eczematous dermatitis	— (contact dermatitis phenotype considered; etiology unconfirmed)	IV amoxicillin-clavulanate + IV methylprednisolone + saline irrigation (7 days)	Minimal improvement; lesions persisted	Figure 1B
Start oral tacrolimus (Course 1)	Considered rescue therapy due to risk of skin necrosis/framework exposure	—	Oral tacrolimus 1 mg BID ×5 d → 1 mg QD ×5 d → 0.5 mg QD ×3 d → 0.5 mg QOD ×4 d (total 17 days; starting dose ∼0.037 mg/kg/day based on 54 kg)	Improvement noted by tacrolimus day 5; complete clearance by day 13; no dyspigmentation/scaling/scarring	Figure 1C
Monitoring (Course 1)	Safety monitoring	—	BP + renal/hepatic function on tacrolimus day 5 and day 10: normal; trough levels not measured	No adverse or unexpected events	—
July 5, 2024	Stage II auricular reconstruction	Intraoperative antisepsis (povidone-iodine)	Standard postoperative care	—	—
POD 10 (suture removal)	Recurrence of eczema around reconstructed auricle and abdominal donor site; erythema/vesicles/pruritus; no systemic symptoms	Povidone-iodine used for disinfection at suture removal (temporal association)	Saline cleansing + oral loratadine + topical mometasone furoate + fusidic acid (∼3 days)	Minimal improvement	Figure 2A–C
Restart oral tacrolimus (Course 2)	Refractory postoperative dermatitis (similar phenotype at two postoperative fields)	—	Oral tacrolimus restarted at 1 mg BID, same total duration and tapering schedule as Course 1 (total 17 days)	Noticeable improvement by tacrolimus day 3; complete clearance by day 10	Figure 3A
Monitoring (Course 2)	Safety monitoring	—	BP + renal/hepatic function on tacrolimus day 5 and day 10: normal; trough levels not measured	No infections; no adverse or unexpected events	—
3 months follow-up	No recurrence; reconstructed auricle with excellent aesthetic appearance; no pigmentary/textural sequelae	Avoidance/alternative antiseptic strategy (if implemented)	—	Stable outcome	Figure 3B,C

## Discussion

Congenital microtia represents one of the most common craniofacial birth defects in children, with a reported incidence ranging from 1:1,000 to 1:10,000. The condition predominantly affects the right side and occurs approximately 30% more frequently in boys than in girls (4). Autologous rib cartilage total auricular reconstruction remains the gold-standard surgical approach (5). The two predominant techniques are the Nagata method (6) and the tissue-expansion method (7). Recognized complications of rib cartilage ear reconstruction include hematoma, infection, flap ischemia and necrosis, cartilage framework resorption or exposure, and eczematous dermatitis. Because the reconstructed auricle consists of a thin skin envelope directly overlying the cartilage framework with minimal intervening subcutaneous tissue, postoperative eczematous dermatitis, if inadequately controlled, can rapidly progress to secondary bacterial infection, skin necrosis, framework exposure, and unfavorable scarring—all of which severely compromise the final aesthetic outcome. Despite the clinical significance of this complication, published literature specifically addressing post-reconstruction eczematous dermatitis remains limited.

Tacrolimus (8) is a macrolide immunosuppressant that exerts its primary effect by inhibiting calcineurin, thereby blocking interleukin-2 production and subsequent T-lymphocyte proliferation. Initially developed for prevention of rejection following solid-organ transplantation, it has gained increasing off-label use in dermatology in recent years, demonstrating notable efficacy in the management of refractory or inflammatory dermatoses (9)^.^

Current evidence indicates no statistically significant difference in the incidence of postoperative eczematous dermatitis between the Nagata technique and the tissue-expansion method (10). In the present case, povidone-iodine was used intraoperatively as the antiseptic agent, and both episodes of eczematous dermatitis occurred immediately following the first postoperative exposure to povidone-iodine. Taken together, the clinical course is most consistent with postoperative eczematous dermatitis (contact dermatitis on compromised postoperative skin), with povidone-iodine considered a suspected trigger based on reproducible temporal association. A delayed-type hypersensitivity mechanism is plausible; however, irritant dermatitis, an atopic flare, secondary infection/impetiginization, and reactions to dressings or topical agents cannot be excluded in the absence of confirmatory testing. Povidone-iodine is a complex of polyvinylpyrrolidone and elemental iodine. The majority of iodine molecules (99.96%) remain bound within the helical structure of povidone and are released gradually, whereas only a minor fraction (0.04%) exists as free diatomic iodine. Cutaneous reactions temporally associated with povidone–iodine have been reported for decades; a recent systematic review identified 223 patients across 38 eligible studies describing povidone-iodine-associated contact dermatitis, most commonly irritant contact dermatitis followed by allergic contact dermatitis (11). Whether iodine or the povidone carrier is the primary sensitizing agent remains unresolved; however, alcohol-based skin tests are consistently negative in these patients, supporting the use of alcohol-based antiseptics as safe alternatives (12). Upon follow-up inquiry, the patient's family reported prior uneventful exposure to povidone-iodine at other body sites, suggesting that the combination of impaired venous/lymphatic drainage in the elevated skin envelope and povidone-iodine exposure may have synergistically precipitated eczematous dermatitis in the reconstructed auricle. Standard management of post-reconstruction eczematous dermatitis generally includes systemic antihistamines, H1-receptor antagonists, corticosteroids, and local saline irrigation (3, 10, 13). To date, no reports have specifically addressed cases refractory to these conventional measures. In our patient, conventional regimens failed on two separate occasions. Given the risk of rapid progression to cartilage framework exposure and the undesirable cutaneous atrophy associated with prolonged corticosteroid use, oral tacrolimus was administered.

Tacrolimus exerts potent anti-inflammatory and antiallergic effects through calcineurin inhibition, suppression of T-lymphocyte proliferation, and reduction of proinflammatory cytokine release. A stepped-dose regimen (induction–maintenance–taper) was employed to maximize efficacy while minimizing adverse effects. Complete resolution without recurrence was achieved after both treatment courses. In this single, confounded case, short courses of oral tacrolimus were associated with rapid improvement after failure of conventional measures. While causality cannot be established, oral tacrolimus may be considered as a rescue option in exceptional refractory postoperative eczematous dermatitis when the risk of skin compromise over the cartilage framework is high, provided that careful pediatric monitoring is ensured.

Limitations include the absence of confirmatory diagnostic testing (patch testing/biopsy/microbiology), potential confounding from concurrent therapies, and relatively short follow-up. Therefore, the association with povidone-iodine should be regarded as a diagnostic possibility rather than proven causation, and the role of systemic tacrolimus requires further study.

## Caregiver perspective

The caregiver reported that, after surgery, the child developed marked erythema, oozing, and pruritus at both the reconstructed ear and the abdominal donor site, which disrupted sleep and caused substantial distress for the child and family. The family was particularly worried about wound breakdown, secondary infection, and potential compromise of the auricular reconstruction (including framework exposure). Because multiple topical measures and antimicrobial therapy provided limited relief, the caregiver expressed concerns about the safety and expected benefit of further treatments, and noted that topical applications during the oozing/erosive phase sometimes made it difficult to visually assess day-to-day changes in the eruption, which increased anxiety. After initiation of oral tacrolimus with close follow-up and laboratory monitoring, the lesions gradually improved and resolved; pruritus decreased substantially, and sleep and daily activities returned to normal. The caregiver appreciated the shared decision-making process and careful monitoring, and hoped that sharing this case may help other children with similar postoperative inflammatory reactions receive timely and appropriate management.

## Conclusion

This case highlights oral tacrolimus as a potentially effective treatment for refractory postoperative eczematous dermatitis following total auricular reconstruction in pediatric patients. Further studies are warranted to confirm its safety and efficacy in larger cohorts.

## Data Availability

The original contributions presented in the study are included in the article/Supplementary Material, further inquiries can be directed to the corresponding author/s.
